# Regional Crosstalk Between the Amygdala, Hippocampus, and Prefrontal Cortex Following Na^+^,K^+^-ATPase Inhibition by Ouabain

**DOI:** 10.1007/s12640-026-00800-7

**Published:** 2026-05-07

**Authors:** Bianca Estefani Schmidt, Ana Karla Oliveira Leite, Clarissa Penha Farias, Alessandra Schmitt Rieder, Gustavo Ricardo Krupp Prauchner, Carlos Alexandre Netto, Angela T. S. Wyse

**Affiliations:** 1 Postgraduate Program in Translational Neuroscience (PGNeT), National Institute of Translational Neuroscience, Universidade Federal do Rio de Janeiro (UFRJ), Rio de Janeiro, RJ Brazil; 2 Neuroprotection and Neurometabolic Diseases Laboratory (Wyse’s Lab), Department of Biochemistry, Universidade Federal do Rio Grande do Sul (UFRGS), Porto Alegre, RS Brazil; 3 Department of Biochemistry, Instituto de Ciências Básicas da Saúde (ICBS), Universidade Federal do Rio Grande do Sul, Porto Alegre, RS Brazil

**Keywords:** Na^+^K^+^-ATPase activity, Fear memory consolidation, Ouabain, Oxidative stress, Neuroinflammation

## Abstract

The Na⁺,K⁺-ATPase plays a fundamental role in memory formation by regulating neuronal excitability and NMDA receptor-dependent synaptic plasticity. While this enzyme’s dysfunction is broadly linked to cognitive disorders, its specific contribution to fear memory consolidation, particularly within the key brain structure of the basolateral amygdala (BLA), remains undefined. We investigate whether Na^+^,K^+^-ATPase inhibition disrupts contextual fear conditioning (CFC) memory consolidation and examine downstream molecular, oxidative, and inflammatory responses in interconnected brain regions. Fifty-day-old male Wistar rats received bilateral cerebral infusions of the Na^+^,K^+^-ATPase inhibitor ouabain (1 μm) or vehicle immediately after CFC training. Memory retention was assessed 24 h later. Additional cohorts were euthanized 6–24 h after infusion for biochemical analysis of Na^+^,K^+^-ATPase activity, expression of Na^+^,K^+^-ATPase isoforms (α1, α2, α3, β1), oxidative stress markers, and pro-inflammatory cytokines in the amygdala, hippocampus, and prefrontal cortex. Post-training Na^+^,K^+^-ATPase inhibition in the BLA selectively impaired fear memory consolidation. Ouabain induced changes in Na⁺,K⁺-ATPase activity in the hippocampus and prefrontal cortex, and induces time-dependent upregulation of α1 and β1 isoforms. Six hours after infusion, ouabain triggered oxidative imbalance – characterized by reduced CAT activity. Pro-inflammatory cytokine expression was markedly elevated in the hippocampus and prefrontal cortex, while D-serine co-administration failed to rescue memory deficits. These findings establish a previously unrecognized role for Na⁺,K⁺-ATPase signaling in the BLA in coordinating the neurobiological processes essential for fear memory consolidation, indicating that its precise regulation is required for adaptive fear memory formation.

**Supplementary Information:**

The online version contains supplementary material available at 10.1007/s12640-026-00800-7.

## Introduction

The Na^+^,K^+^-ATPase is a ubiquitous membrane-bound enzyme that plays a central role in neuronal function and cellular homeostasis. By actively transporting sodium (Na^+^) out of the cell and potassium (K^+^) into the cell, the enzyme maintains the electrochemical gradients essential for neuronal excitability, neurotransmitter uptake, and synaptic transmission (Kaplan 2002; Sweadner 1989). In the brain, Na^+^,K^+^-ATPase accounts for nearly 50% of total energy consumption (Harris et al. 2012), highlighting its importance in high-demand processes such as learning and memory (Wyse et al. 2004). Beyond ion pumping, Na^+^,K^+^-ATPase functions as a signaling molecule that regulates intracellular cascades involved in cell growth, differentiation, apoptosis, and inflammation (Aperia 2007; Xie 2003).

The functional diversity of the pump is reflected in its complex molecular composition. Structurally, Na^+^,K^+^-ATPase is composed of α (catalytic), β (glycoprotein), and FXYD (regulatory) subunits. Multiple isoforms of each subunit display region-specific expression and distinct regulatory properties (Blanco and Mercer 1998; Geering 2008). In the central nervous system (CNS), the α1 isoform predominates as the housekeeping variant, whereas α2 and α3 show more restricted expression patterns and are strongly associated with neuronal excitability (Dobretsov 2005; Lytton 1985). The β1 isoform is the most abundant in the brain and is crucial for proper enzyme assembly and membrane stability (Geering 2001).

A powerful tool for probing this enzyme’s functions is ouabain, a specific inhibitor that also acts as an endogenous modulator. Ouabain, a cardiac glycoside originally isolated from *Strophanthus gratus*, also occurs endogenously in mammals and binds with high affinity to the α-subunit of Na⁺,K⁺-ATPase, inhibiting its activity and disrupting ionic homeostasis (Lingrel and Kuntzweiler 1994; Laredo et al. 1995). Importantly, by binding to the pump, ouabain does not only block ion transport but also initiates specific signaling cascades. In addition to its canonical role in maintaining transmembrane Na⁺ and K⁺ gradients, Na⁺,K⁺-ATPase functions as a signal transducer capable of activating intracellular pathways involved in neuronal excitability and synaptic regulation, including calcium-dependent and ERK/CREB-related cascades (de Lores Arnaiz & Ordieres, 2014; Xie and Askari 2002). Consistent with this, intracerebral administration of ouabain has been shown to impair learning and memory performance and to modulate signaling pathways that regulate the expression and function of plasticity-related molecules, such as brain-derived neurotrophic factor (BDNF), which plays a well-established role in memory consolidation (Bekinschtein et al. 2008; Valvassori et al. 2021).

The pivotal role of Na⁺,K⁺-ATPase in memory is particularly evident in the regulation of synaptic plasticity, where it counteracts the ionic fluxes generated by N-methyl-D-aspartate (NMDA) receptor activation (Arnaiz and Bersier 2014). More specifically, the involvement of Na⁺,K⁺-ATPase in memory formation is closely linked to the induction of synaptic plasticity mediated by NMDA receptors. Glutamate binding to the NMDA receptor is insufficient for its activation. Full channel opening and subsequent calcium influx require the simultaneous binding of a co-agonist to the glycine modulatory site located on the GluN1 subunit. D-serine (an endogenous gliotransmitter and obligatory NMDA receptor co-agonist) binds to this site, acting as a necessary key to “unlock” the receptor for glutamate (Wolosker and Balu 2020). Consequently, full NMDA receptor activation triggers substantial Na⁺ and Ca²⁺ influxes, disrupting ionic homeostasis and thus necessitating enhanced Na⁺,K⁺-ATPase activity to restore the resting equilibrium (Bliss and Collingridge 2013; Akkuratov et al. 2015). This homeostatic restoration is critical for memory consolidation, a process that depends on subsequent gene transcription, protein synthesis, and long-term neuronal reorganization (Squire 1986; Abel and Lattal 2001; Guzmán-Ramos 2011; Izquierdo et al. 2016), processes which are highly sensitive to perturbations in ionic and metabolic balance.

To study these mechanisms, contextual fear memory provides an excellent model due to its robust neural circuitry and clinical relevance. Fear memory is particularly relevant because of its high survival value and its well-established involvement in anxiety disorders, phobias, and post-traumatic stress disorder (LeDoux 2000). The basolateral amygdala (BLA) is a central hub in fear learning and consolidation, integrating sensory information and coordinating emotional memory processing with the hippocampus and prefrontal (Rodrigues et al. 2004; Sah et al. 2003). The hippocampus is critically involved in encoding the contextual and spatial components of fear memories, enabling discrimination between safe and threatening environments (Pitkänen et al. 2000). In parallel, the medial prefrontal cortex contributes to the regulation and expression of fear responses through top-down modulation of amygdala activity (Milad and Quirk 2012; Quirk and Beer 2006).

The function of this memory circuitry can be profoundly influenced by the brain’s redox state. Oxidative stress is a major modulator of Na⁺,K⁺-ATPase activity in the CNS. Excessive reactive oxygen species (ROS) oxidize the α-subunit, diminishing enzymatic activity and contributing to intracellular Na⁺ accumulation, Ca²⁺ overload, mitochondrial dysfunction, and excitotoxicity – all central events in neurodegenerative and neuroinflammatory processes (Liu et al. 2020). The Na⁺,K⁺-ATPase/Src complex also participates in redox-sensitive signaling that activates MAPK/ERK and NF-κB pathways, influencing neuronal survival and synaptic plasticity (Liu et al. 2018). Antioxidant defenses can mitigate these effects (Yan et al. 2017).

Closely intertwined with oxidative stress, neuroinflammation represents another critical pathway influenced by Na^+^,K^+^-ATPase dysfunction. Pro-inflammatory cytokines modulate enzyme expression and activity, while impaired Na^+^,K^+^-ATPase may intensify inflammatory responses (Kinoshita et al. 2014; Moseley et al. 2003). Ouabain itself can bidirectionally modulate nuclear factor-kappa B (NF-κB) and IL-1β expression depending on dose and cell type (Cavalcante-Silva et al. 2017), and its pro-inflammatory effects can involve tumor necrosis factor receptor 1 (TNFR1) signaling (Kinoshita et al. 2022). Cytokines such as TNF-α and IL-1β, acting through TNFR1 and NF-κB activation, are central mediators of neuroinflammation and can profoundly affect memory processing (Dinarello 2011; Hayden and Ghosh 2011; Wajant et al. 2003).

Given these interrelated mechanisms, understanding how ouabain-induced Na^+^,K^+^-ATPase dysfunction affects fear memory and triggers oxidative and inflammatory cascades in distinct brain regions is essential for elucidating its role in neurotoxicity. Therefore, the present study investigates the effects of intracerebral ouabain administration on fear memory consolidation, Na^+^,K^+^-ATPase activity and isoform expression, oxidative stress markers, and inflammatory cytokine gene expression in the amygdala, hippocampus, and prefrontal cortex of Wistar rats. This regional analysis aims to clarify the mechanisms by which Na^+^,K^+^-ATPase inhibition contributes to cognitive dysfunction and neurotoxic processes.

## Materials and Methods

### Ethics Statement

All procedures were approved by the Ethics Committee of the Federal University of Rio Grande do Sul, Brazil (protocol nº 40458). Experiments were conducted under veterinarian supervision and in accordance with institutional guidelines, the Brazilian Society of Science in Laboratory Animals, and the Guide for the Care and Use of Laboratory Animals, adopted by the National Institutes of Health (NIH, USA).

### Animals

A total of 101 fifty-days-old male adult Wistar rats (270–330 g) were obtained from the Centro de Reprodução e Experimentação de Animais de Laboratório (Institute of Basic Health Science, Federal University of Rio Grande do Sul, Brazil). Animals were housed four per cage in a temperature-controlled room (22 ± 1 °C) on a 12:12-h light–dark cycle (lights on at 07:00), with food and water available *ad libitum*. A standard commercial rodent diet (Nuvilab CR-1 irradiated chow, Quimtia^®^) was provided. Drinking water was supplied from the local source and filtered before to use; no specific analysis for contaminants was performed.

### Experimental Design

Animals were received at the experimental facility in independent cohorts, in accordance with the experimental design. The first cohort (*n* = 63) was used for behavioral testing to evaluate memory consolidation across three distinct brain regions, including training, infusion, and *ex vivo* analyses performed 24 h after the procedure. The second cohort (*n* = 26) was used to investigate the potential involvement of NMDA receptors. The third cohort (*n* = 12) was dedicated to biochemical and molecular analyses, with animals euthanized 6 h after ouabain infusion. Within each cohort, animals were randomly allocated to experimental groups.

Upon arrival, rats were allowed to habituate to the vivarium for 7 days and then handled for three consecutive days. This was followed by stereotaxic surgery for bilateral cannula implantation, as detailed in Sect.  2.4. Animals recovered for 7 days post-surgery, being pair-housed for the initial 48 h before being reallocated into groups of four per cage. Four days post-surgery, animals were handled for 5 min daily for three days.

#### *In vivo* Phase (Sect.  2.5)

To evaluate the effects of OUA on the consolidation of contextual fear conditioning (CFC), rats submitted to CFC and assigned to receive either vehicle (VEH − 0.9% NaCl) or ouabain (OUA) or ouabain co-administered with D-serine, as detailed in Sect.  2.5.2.

#### *Ex vivo* Phase (Sect.  2.6)

To analyze the Na^+^,K^+^-ATPase activity, isoform-specific gene expression, immunocontent, and oxidative/inflammatory parameters in the BLA, hippocampus, and prefrontal cortex (PFC), rats were euthanized by decapitation and the BLA, hippocampus and PFC were rapidly dissected and immediately stored at -80 °C until further processing. Biochemical and molecular analyses were performed exclusively in animals that did not receive D-serine.

**Fig. 1:**
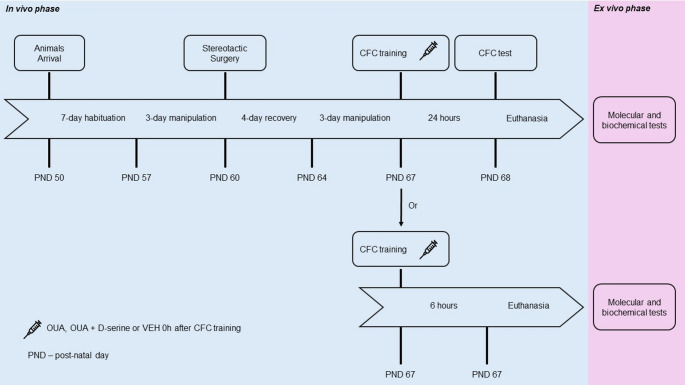
Experimental protocol. Adult male Wistar rats underwent a 7-day habituation period, followed by 3 days of handling to accustom the animals to contact with the researcher. Subsequently, rats were subjected to stereotaxic surgery and allowed a 7-day postoperative period, with the first 4 days dedicated to recovery. From postoperative day 5 onward, animals were handled again until the beginning of the behavioral procedures

The overall experimental timeline is presented in Fig. 1.

### Stereotaxic Surgery

At least one week after arrival, animals were anesthetized (75 mg/kg ketamine plus 10 mg/kg xylazine; intraperitoneal) and placed on a stereotaxic frame. Stainless steel guide cannulae (22 gauge) were bilaterally implanted 1 mm above the BLA (anterior, -2.4 mm; lateral, ± 5.1 mm; ventral, -3.1 mm; from Bregma), the CA1 region of the dorsal hippocampus (anterior, -4.2 mm; lateral, ± 3.0 mm; ventral, -1.8 mm; from Bregma), or the prelimbic cortex (PrL; anterior, + 3.2 mm; lateral, ± 0.8 mm; ventral, -2.0 mm; from Bregma) using coordinates from Paxinos and Watson (Paxinos and Watson 1986). Cannulae were fixed to the skull with dental acrylic. Animals recovered for 7 days before behavioral experiments.

### *In vivo* Phase

#### Contextual Fear Conditioning (CFC)

Animals were individually placed in a conditioning chamber (24 × 25 × 21 cm) with a grid floor for shock delivery. After a 2-min acclimation period, animals received three footshocks (0.5 mA, 2 s) at 30-s interval. Animals were removed from the conditioning chamber 30 s after the last footshock and placed back in their home cages. CFC apparatus was cleaned with 70% ethanol between subjects. Twenty-four hours later, animals were re-exposed to the same apparatus for 3 min without footshocks (Fiorenza et al. 2012). Freezing behavior – defined as complete immobility except for respiratory movements – was scored by a trained observer (Fanselow 1980), expressed in seconds (s), calculated and plotted as %.

The results of the training phase were calculated using the formula:

$$\:Freezing\:\left(s\right)\:\times\:100\div120$$

And of the test phase were calculated using the formula:

$$\:Freezing\:\left(s\right)\:\times\:100\div180$$

#### Drugs and Infusion Procedures

Infusions were always administered bilaterally and restricted to a single target structure per animal. Within 1 min after CFC training, infusion cannula was connected to a Hamilton microsyringe and inserted so that the tip extended 1.0 mm beyond the guide cannula in CA1 and PrL, and 1.4 mm in BLA. Infusions were performed at 1 µL/min (0.5 µL per side into the BLA; 1 µL per side into CA1 and PrL). Infusions lasted 30 s after which the infusion cannula was left in place for an additional 15 s to minimize backflow. Then they were carefully withdrawn and placed on the other side where the procedure was repeated. The entire bilateral infusion procedure took about 90 s. The following drugs were used, at the doses stated in each case: ouabain (Sigma-Aldrich #O3125; St. Louis, MO, U.S.), 1 µM (Riegel et al. 2009); D-serine (Sigma-Aldrich #S4250; St. Louis, MO, U.S.), 50 µg/side (Fiorenza et al. 2012). Both drugs were dissolved in 0.9% wt./vol. NaCl.

#### Cannula Placements Verification

**Fig. 2:**
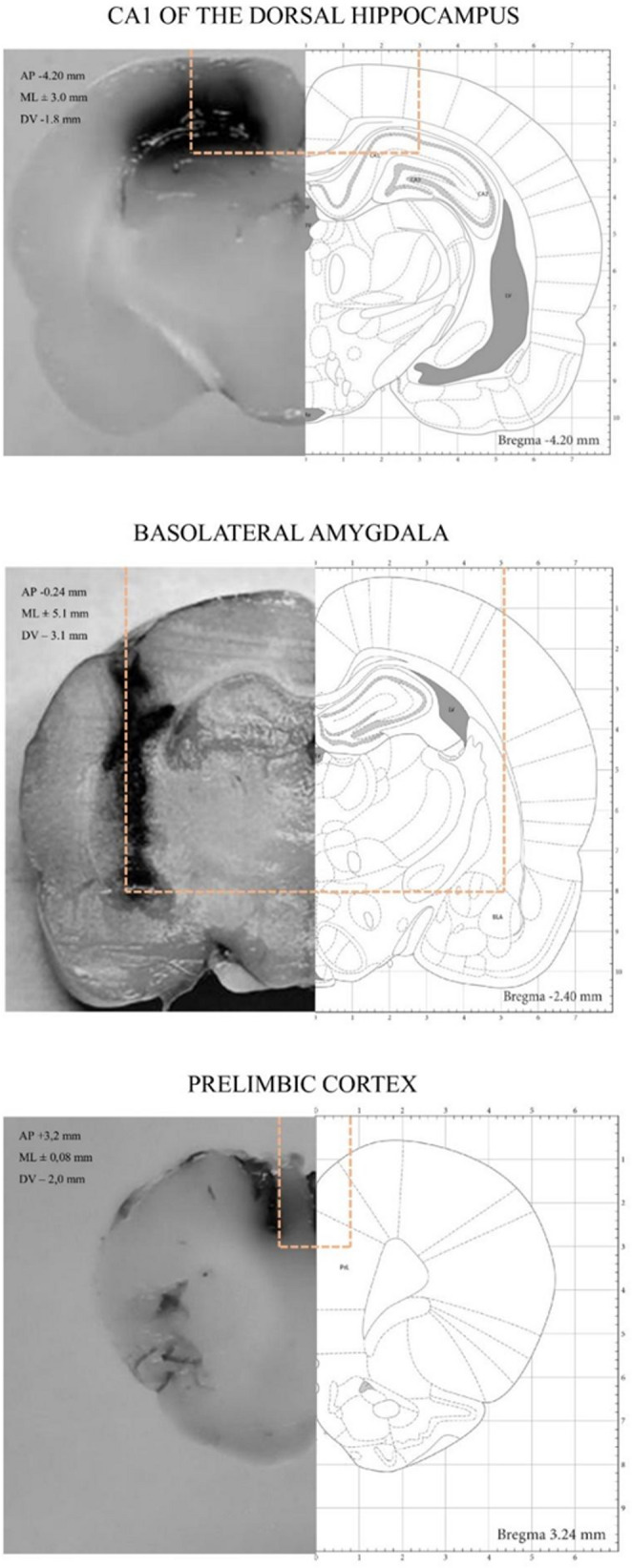
Representative guide cannula placement adapted from Paxinos and Watson (2009)

Cannula placement was verified in a subset of animals (*n* = 3; one per target brain region) to ensure accurate tissue collection for subsequent biochemical analyses. Animals received an infusion of 4% methylene blue (1.0 µL in CA1 and PrL; 0.5 µL in BLA) and were euthanized by decapitation 15 min later. Cannula placement was considered accurate when dye dispersion was observed within approximately 1.0 mm³ of the intended infusion site. Animals with placements outside this range were excluded from the analyses. Cannula placement verification is illustrated in Fig. 2.

### *Ex vivo* Phase

Animals were implanted with bilateral BLA cannulae and assigned to either vehicle or OUA groups (*n* = 4–6 animals per group) (Dos Santos et al. 2021). For biochemical and molecular analyses, animals were randomly assigned to euthanasia by decapitation without anesthesia at either 6–24 h after infusion.

#### Tissue Collection and Processing

Each animal was marked with a unique code to track the collected tissues and ensure accurate assignment to endpoints. Tissues were dissected on ice immediately after euthanasia, and the right and left portions of each brain region were collected separately, placed in individual tubes, and stored at − 80 °C. To avoid potential lateralization bias, right and left samples were randomly assigned to different experimental endpoints.

For analysis, samples were thawed on ice, weighed and divided into aliquots that were randomly allocated to each experimental technique. Each aliquot was then independently processed and homogenized in assay-specific buffers, as described below. All homogenates were maintained on ice throughout processing to minimize enzymatic degradation and preserve molecular integrity.

#### Na^+^,K^+^-ATPase Activity

Tissues were homogenized (1:10, w/v) in 0.32 mM sucrose containing 5.0 mM HEPES (Sigma-Aldrich #H3375; St. Louis, MO, U.S.) and 1.0 mM EDTA (pH = 7.5), followed by centrifugation at 1000 x g for 10 min at 4 °C. An aliquot of 10 µL of the resulting supernatants was incubated in a reaction medium containing (in mM): 80 NaCl (NEON #01021; Suzano, SP, Brazil), 20 KCl (NEON #01005; Suzano, SP, Brazil), 5 MgCl_2_ (NEON #00976; Suzano, SP, Brazil), and 40 Tris–HCl pH 7.5 (Sigma Aldrich # 1503; St Louis, U. S.) (pH 7.4). After 10-min pre-incubation at 37 °C, the reaction was initiated by adding 3.0 mM ATP (Sigma-Aldrich #A2383; St. Louis, MO, U.S.) and allowed to proceed for 20 min. Parallel incubations containing 1 mM ouabain (Sigma-Aldrich #O3125; St. Louis, MO, U.S.) were used to determine nonspecific ATPase activity. Na^+^/K^+^-ATPase activity was calculated as the ouabain-sensitive fraction (De Souza Wyse et al. 2000). Inorganic phosphate (Pi) released was quantified according to Chan et al. (Chan et al. 1986), and enzymatic activity was expressed as nmol Pi/min/mg protein.

#### Quantitative Real-time PCR

Total RNA was isolated from the amygdala, hippocampus and prefrontal cortex using TRIzol^®^ reagent (Thermo Fisher Scientific, Rockford, IL, USA). The concentration and purity of the RNA were determined spectrophotometrically at a ratio of 260:280. Extracted RNA (1 µg) was submitted to cDNA synthesis by High-Capacity cDNA Reverse Transcription Kit (Applied Biosystems, Thermo Fisher Scientific #436813; Massachusetts, U.S.) in 20 µL reaction according to the manufacturer’s instructions. Gene expression levels of *Tnfr1* (#Rn01492348_m1), *nfκb1* (#Rn01399572_m1), *il1b* (#Rn00580432_m1), and Na^+^,K^+^-ATPase isoforms (*Atp1a1*, #Rn01533986_m1; *Atp1a2*, #Rn00560789_m1; *Atp1a3*, #Rn00560813_m1; *Atp1b1*, #Rn00565405_m1) and the reference gene *Actb* (#Rn00667869_m1) were quantified using the TaqMan^®^ real-time RT-PCR assay with inventoried primers and probes from Applied Biosystems. All reactions were run for a maximum of 40 cycles, and this value was used as the upper threshold for data acceptance. Target gene expression was normalized to β-actin, and relative mRNA levels were calculated using the 2 − ΔΔCt method (Livak and Schmittgen 2001).

#### Western Blotting

Cerebral tissues were homogenized in lysis solution (2 mM EDTA, 50 mM tris-hydrochloric acid (HCl), pH 6.8, and 4% SDS). Then, samples were dissolved 1:1 in Laemmli buffer 2 × containing 40% glycerol, 5% 2-mercaptoethanol, 50 mM tris–HCl, pH 6.8, and 10% SDS and boiled for 5 min. Total protein homogenates (4–6 animals per group) were separated on 10% SDS–PAGE gels (40 µg protein per lane) and transferred to nitrocellulose membranes using a semi-dry transfer system (Trans-Blot SD, Bio-Rad; 1 h at 15 V) in transfer buffer containing 48 mM Tris, 39 mM glycine (Sigma Aldrich #G8898; St Louis, U.S.), 20% methanol (NEON #00495; Suzano, SP, Brazil), and 0.25% SDS (Dinamica #1587; Indaiatuba, SP, Brazil). Membranes were washed for 10 min in Tris-buffered saline (TBS; 0.5 M NaCl, 20 mM Tris [Sigma Aldrich #T1503; St Louis, U. S.], pH 7.5) and blocked for 2 h in TBS with 0.05% Tween-20 (Sigma Aldrich #P1379; St Louis, U. S.) (TTBS). Membranes were incubated overnight at 4 °C with the following primary antibodies diluted in TTBS: mouse anti-Na^+^,K^+^-ATPase α1 isoform (1:10.000, Sigma-Aldrich #ZMS1029); rabbit anti-Na^+^,K^+^-ATPase α2 (1:500, Sigma-Aldrich #SAB5701244); mouse anti-Na^+^,K^+^-ATPase α3 isoform (1:1.000, Sigma-Aldrich #A273); goat anti-Na^+^,K^+^-ATPase β1 (1:2.000, Sigma-Aldrich SAB2501588) or anti-β-actin (1:1.000, Sigma-Aldrich #A1978). After two washes in TTBS (5 min each), membranes were incubated for 2 h at room temperature with the appropriate HRP-conjugated secondary antibodies: anti-mouse IgG (1:1.000, Cell Signaling Technology 7076), anti-rabbit IgG (1:1000, Cell Signaling Technology #7074), or anti-goat IgG (1:5000, Merck #AP106P). Blots were then washed twice in TTBS (5 min each) and twice in TBS. Protein bands were visualized using an enhanced chemiluminescence substrate (Immobilon Western Chemiluminescent HRP Substrate, Millipore, #34578; St Louis, U.S.) and imaged with an ImageQuant LAS 4000 system (GE Healthcare). Molecular weight markers were determined using a prestained protein ladder (Pierce™ Unstained Protein Molecular Weight Marker, Thermo Fisher Scientific, #26616; Waltham, MA, U.S.). After detection of target proteins, membranes were stripped and reprobed with an anti-β-actin antibody. Band intensities were quantified using ImageJ (NIH), and target protein levels were normalized to the corresponding β-actin signal from the same lane. Results were expressed as a percentage of control (Ramires Junior et al. 2022). Full, uncropped western blot images are provided in the Supplementary Material.

#### Oxidative Stress Parameters Assays

For evaluation of redox homeostasis parameters, the cerebral cortex was homogenized (1:10, w/v) in 140 mM potassium chloride and 20 mM sodium phosphate buffer, pH 7.4. The homogenates were centrifuged at 3000 rpm for 10 min at 4 °C. The supernatant was immediately collected and used for subsequent analyses.

##### Nitrite Levels

Nitrite levels were quantified using the Griess reaction. A 50 µL aliquot of sample supernatants was incubated for 10 min at room temperature with Griess reagent prepared as a 1:1 mixture of 1% sulfanilamide (Sigma-Aldrich, S9251; St. Louis, USA) in 5% phosphoric acid and 0.1% N-(1-naphthyl)ethylenediamine dihydrochloride (Sigma- Aldrich #N9125; St. Louis, USA). Nitrite concentrations were calculated based on a sodium nitrite standard calibration curve, and results were expressed as micromoles of nitrite per milligram of protein (Green et al. 1982).

##### 2′7′- Dichlorofluorescein (DCF) Fluorescence Assay

Reactive species formation was assessed using the method described by LeBel et al. (1992). A 60 µL aliquot of tissue homogenate was incubated with 100 µM 2′7′- dichlorofluorescein diacetate (H2DCF-DA) (Sigma-Aldrich #D6883; St. Louis, U.S.). Following intracellular deacetylation and subsequent oxidation of H2DF, the fluorescent product dichlorofluorescein (DCF) was generated. Fluorescence was measured at an excitation wavelength of 488 nm and emission wavelength of 525 nm. Results were expressed as nanomoles of DCF per milligram of protein (LeBel et al. 1992).

##### Sulfhydryl Content

Sulfhydryl groups were quantified using the method described by Aksenov & Markesbery (2001). This assay is based on the reduction of 5,5′-dithiobis-2-nitrobenzoic acid (DTNB) (Sigma Aldrich #D8130; St Louis, U. S.) by free sulfhydryl groups, producing the yellow-colored derivative 5-thio-2-nitrobenzoic acid (TNB), which is measured spectrophotometrically at 412 nm. Briefly, 15 uL of homogenate were added to 275 uL of phosphate-buffered saline (pH 7.4) containing 1 mM EDTA (Sigma-Aldrich #E9884; St. Louis, MO, U.S.). The reaction was initiated by adding 10 μm DTNB, followed by incubation for 30 min at room temperature in the dark. Sulfhydryl content is inversely related to protein oxidative damage. Results were expresses as nanomoles of TNB per milligram of protein.

##### Superoxide Dismutase (SOD) Activity

Superoxide dismutase (SOD) activity was determined according to the method described by Marklund and Marklund (1974), which is based on the autoxidation of pyrogallol (Sigma Aldrich #P0381; St Louis, U.S), which is inhibited by SOD. For this assay, 15 µL of tissue homogenate were added to the reaction system. The rate of inhibition was monitored spectrophotometrically at 420 nm. A standard curve was generated using purified SOD to calculate enzyme activity in the sample. One unit of SOD activity was defined as the amount of enzyme required to inhibit 50% of pyrogallol autoxidation. Results were expressed as SOD units per milligram of protein.

##### Catalase (CAT) Activity

CAT activity was quantified according to Aebi (1984), which measures the rate of hydrogen peroxide (H2O2) decomposition. An aliquot of 10 µL of tissue was mixed with 0.1% Triton X-100 (Sigma Aldrich #V900502; St Louis, U.S.) and preincubated for 15 min at room temperature. The reaction was initiated by adding 10 mM potassium phosphate buffer (pH 7.0) containing 20 mM H2O2 (Neon #01984; Suzano, SP, Brazil). The decrease in H2O2 absorbance was monitored spectrophotometrically at 240 nm over a period of 3–4 min. One unit of CAT activity was defined as the amount of enzyme that decomposes 1 mmol of H2O2 per minute. Specific activity was expressed as CAT units per milligram of protein.

#### Protein Quantification

Protein concentrations were measured using either the Bradford (Bradford 1976) or Lowry method (Lowry et al. 1951), depending on the assay, with sample volumes of 20 µL and 10 µL, respectively. Bovine serum albumin (Sigma-Aldrich #A7906; St. Louis, U.S.) was used as the standard in both protocols.

### Statistical Analysis

Statistical analysis was performed using GraphPad Prism 8.0.1. Outliers were removed using the ROUT test (Q = 10%). Behavioral data were analyzed using two-way ANOVA followed by Tukey’s post hoc test. Biochemical and molecular data were analyzed using Student’s t-test after verification of normality (Shapiro–Wilk test). Data are expressed as mean ± SEM, with significance set at *p* < 0.05. Sample sizes are indicated in figure legends.

## Results

### Behavioral Assessments

The experiment aimed to assess the role of Na^+^,K^+^-ATPase activity in fear memory consolidation. Animals were trained in the contextual fear conditioning (CFC) task and received (0 h after) bilateral infusions of ouabain (1 µM) or saline into the BLA, prelimbic cortex (PrL), or CA1 region of the hippocampus. Freezing behavior was measured 24 h later during a CFC test session.

**Fig. 3:**
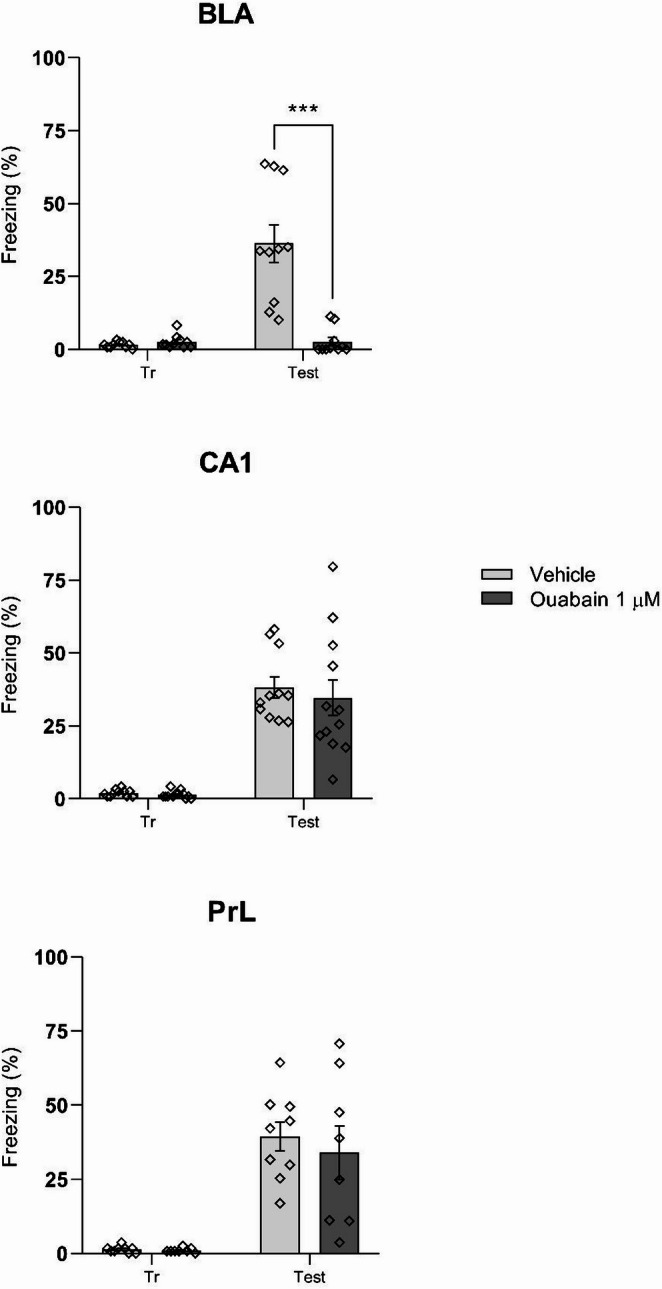
Inhibition of Na^+^,K^+^-ATPase in the BLA impairs consolidation of context-conditioned fear memory. Animals underwent CFC (three 2-s, 0.5-mA scramble footshocks separated by 30-s intervals) and immediately received bilateral infusions of vehicle (0.9% NaCl) or ouabain (1 µM) into the BLA (0.5 µL/side), CA1 or PrL (both 1 uL/side). Twenty-four hours later, rats were tested in a 3-min retention session. The percentage of time spent freezing during the test session is shown. Bilateral infusion of ouabain into de BLA, but not the CA1 or PrL, impaired fear memory consolidation. Four outliers were identified and removed prior to statistical analysis. Data are expressed as mean ± SEM (*n* = 8–12/group). Statistical analysis was performed using two-way ANOVA followed by Tukey’s post hoc test. Across all brain regions and treatment conditions (vehicle and ouabain). *p* < 0.05 were considered statistically significant

As shown in Fig. 3, ouabain infusion into the BLA significantly impaired fear memory consolidation [F (1,9) = 26,06, *p* = 0.0001], indicated by reduced freezing behavior during the test session. No significant effects were observed following ouabain infusion into the PrL or CA1 region of the hippocampus (*p* > 0.05).

**Fig. 4:**
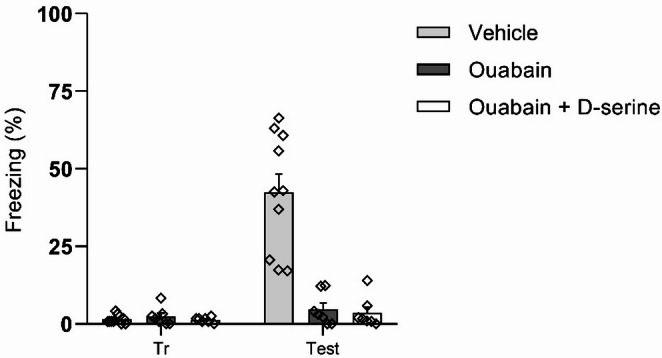
The impairment in fear memory consolidation induced by ouabain is not reversed by D-serine. Animals were subjected to CFC and immediately received bilateral infusions of vehicle (0.9% NaCl), ouabain (1 µM; 0.5 µL/side), or ouabain plus D-serine (50 µg/side) into the BLA. A 3-min retention test was performed 24 h later. D-serine did not reverse the impairment in freezing behavior induced by ouabain. Three outliers were identified and removed prior to statistical analysis. Data are expressed as mean ± SEM (*n* = 7–10/group). Statistical analysis was performed using two-way ANOVA followed by Tukey’s post hoc test. *p* < 0.05 were considered statistically significant

A second experiment was conducted using only BLA-implanted animals. Immediately after CFC training, animals received infusions of saline, ouabain (0.5µL/side), or ouabain co-administered with D-serine (0.25µL + 0.25µL/side). As shown in Fig. 4, D-serine did not prevent ouabain-induced memory impairment (*p* > 0.05).

### Na^+^,K^+^-ATPase Activity and Expression Analyses

**Fig. 5:**
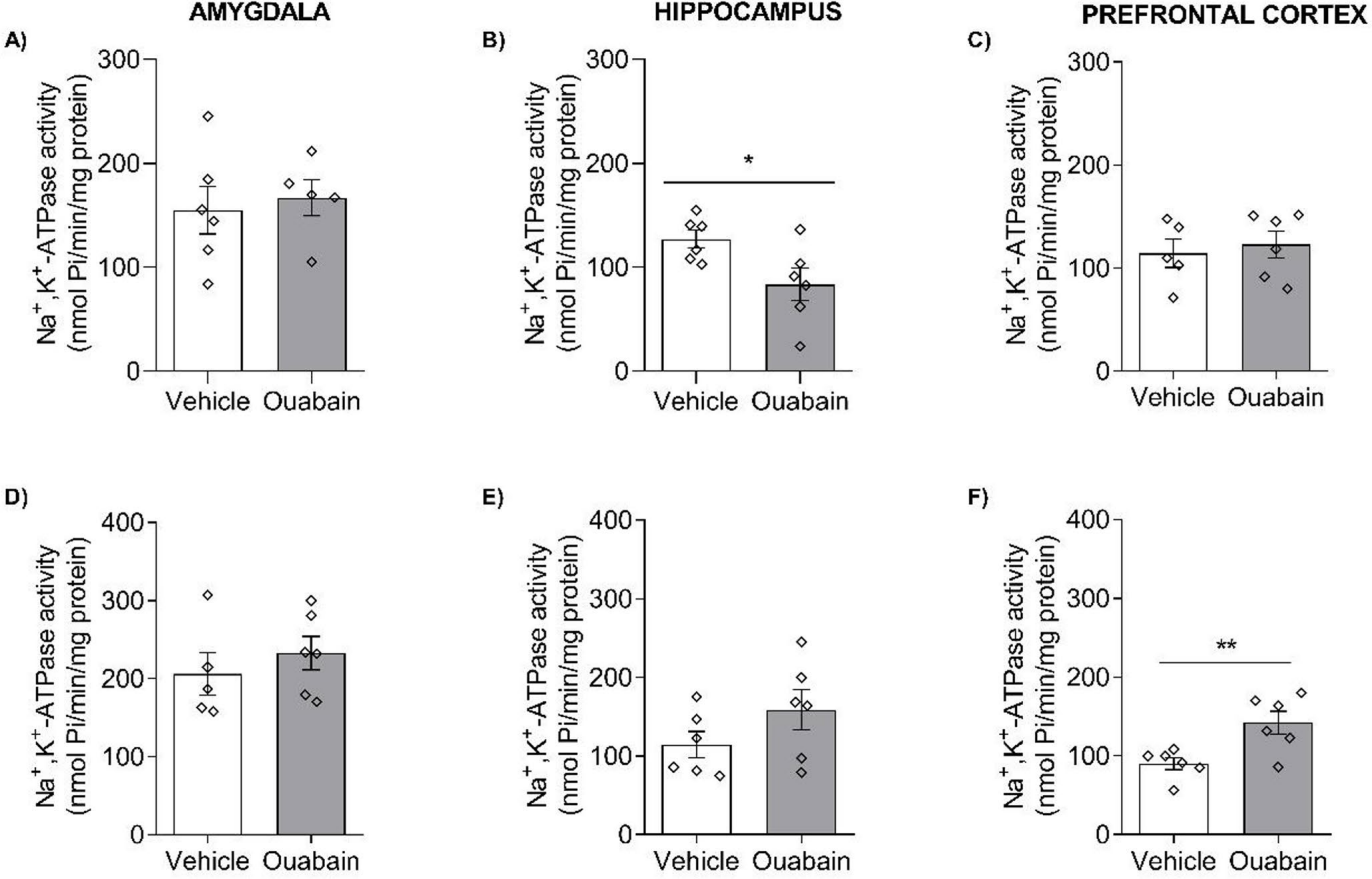
Ouabain infusion into the BLA modulates Na^+^,K^+^-ATPase activity in the hippocampus and prefrontal cortex. Animals underwent CFC and immediately received bilateral infusions of vehicle (0.9% NaCl) or ouabain (1 µM; 0.5 µL/side) into the BLA. Rats were euthanized 6 h (**A**-**C**) or 24 h (**D**-**F**) after infusion, and the amygdala, hippocampus, and prefrontal cortex were dissected for analysis of Na^+^,K^+^-ATPase activity. One outlier was removed from the 24-hour cohort. Data are expressed as mean ± SEM (*n* = 5–6/group). Unpaired *t*-test. *p* < 0.05 were considered statistically significant

To investigate potential associations between ouabain-induced behavioral effects and Na^+^,K^+^-ATPase activity modulation, a separate cohort of rats underwent contextual fear conditioning training and was euthanized 6 h after BLA infusion for enzymatic activity measurement. Na^+^,K^+^-ATPase activity was measured in the hippocampus, amygdala, and prefrontal cortex following ouabain infusion (Fig. 5A-C). In the hippocampus, activity was significantly lower in the ouabain-treated group compared to the control group (t = 2.468, df = 10, *p* = 0.0332). No statistically significant differences in activity were observed in the amygdala or prefrontal cortex (*p* > 0.05). Activity was also measured 24 h post-infusion (Fig. 5D-F). At this time point, no significant differences were detected in the hippocampus or amygdala (*p* > 0.05). In the prefrontal cortex, however, activity was significantly higher in the ouabain-treated group compared to the control (t = 3,193, df = 10, *p* = 0,0096).

**Fig. 6:**
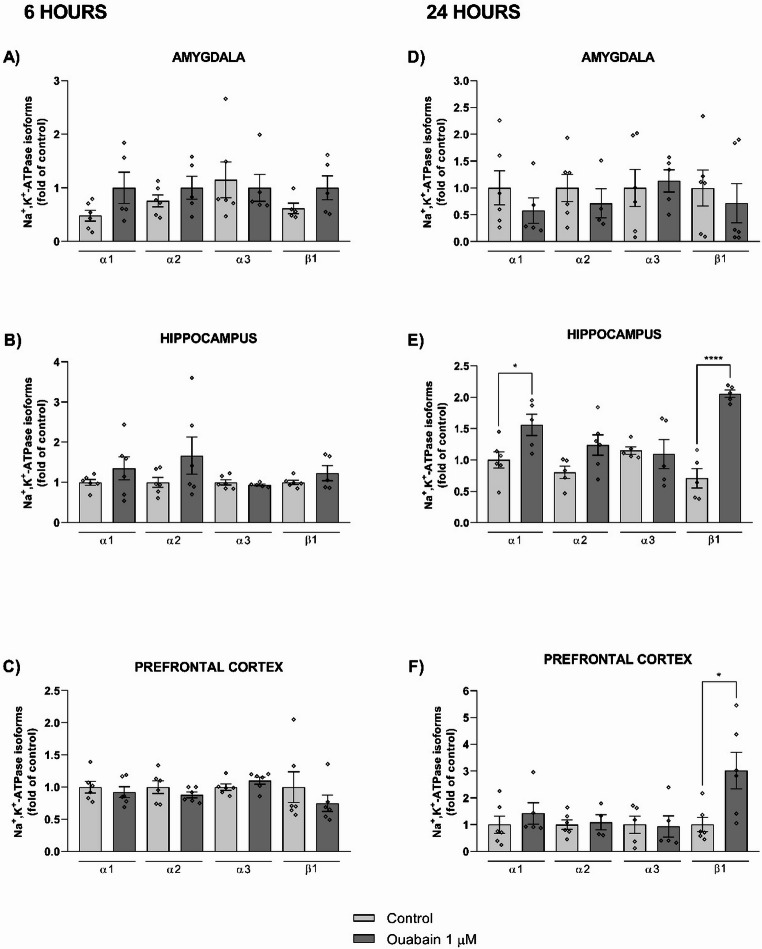
Lack of changes in Na^+^,K^+^-ATPase isoform gene expression 6–24 h later ouabain infusion into the BLA. Animals were subjected to CFC and immediately received bilateral infusions of vehicle (0.9% NaCl) or ouabain (1 µM; 0.5 µL/side) into the BLA. Rats were euthanized and the amygdala, hippocampus, and prefrontal cortex were dissected for quantification of α1, α2, α3 and β1 mRNA expression by RT-qPCR. Six hours later (**A**-**C**), Ct values of control samples ranged from 20.27 to 22.30 (α1), 20.89 to 21.97 (α2), 18.95 to 21.05 (α3), and 18.24 to 19.06 (β1) in the amygdala; from 24.88 to 27.67 (α1), 25.48 to 27.79 (α2), 24.18 to 26.80 (α3), and 22.65 to 25.41 (β1) in the hippocampus; and from 22.91 to 27.51 (α1), 22.67 to 23.97 (α2), 20.35 to 22.97 (α3), and 22.29 to 26.08 (β1) in the prefrontal cortex. Across all structures and isoforms, six amplifications were unsuccessful; among those that amplified, one outlier was excluded (0,7%). Twenty-four hours later (**D**-**F**), Ct values of control samples ranged from 22.46 to 35.52 (α1), 22.67 to 23.97 (α2), 20.35 to 22.97 (α3), and 25.86 to 32.26 (β1) in the amygdala; from 21.33 to 23.09 (α1), 22.67 to 23.97 (α2), 20.35 to 22.97 (α3), and 16.98 to 19.80 (β1) in the hippocampus; and from 26.71 to 34.41 (α1), 28.91 to 35.72 (α2), 27.06 to 34.64 (α3), and 24.79 to 31.54 (β1) in the prefrontal cortex. Across all structures and isoforms, seven amplifications were unsuccessful; among those that amplified, eight outliers were excluded (5,8%). Data were expressed as mean ± SEM (*n* = 4–6/group). Unpaired *t*-test. *p* < 0.05 were considered statistically significant

Gene expression levels of Na^+^,K^+^-ATPase isoforms were analyzed in the hippocampus, prefrontal cortex, and amygdala, as shown in Fig. 6. Six hours after infusion (Fig. 6A-C), no statistically significant differences in mRNA levels were detected for the α1, α2, α3, or β1 isoforms in any of the three brain regions examined (all *p* > 0.05). Based on the observed time course of enzyme activity, gene expression was also assessed at 24 h post-infusion (Fig. 6D-F). At this time point, β1 mRNA levels were increased in the hippocampus (t = 8.213, df = 8, *p* < 0.0001) and prefrontal cortex (t = 2.734, df = 10, *p* = 0.0210), with no significant change observed in the amygdala (*p* > 0.05). An increase in α1 mRNA was also measured in the hippocampus (t = 2.675, df = 9, *p* = 0.0254). No significant changes in mRNA levels were observed for the α2 or α3 isoforms in any brain region at 24 h (all *p* > 0.05). Gene expression of β-actin did not differ between vehicle- and ouabain-treated groups (Fig S9 – supplementary data).

**Fig. 7:**
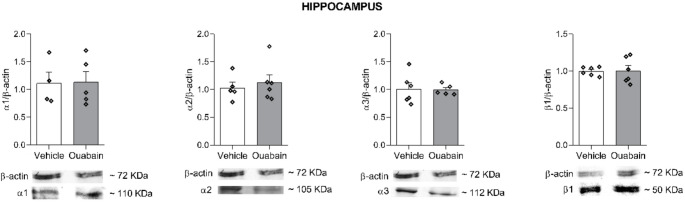
Ouabain infusion into the BLA does not alter Na^+^,K^+^-ATPase isoforms immunocontent in hippocampus. Animals were subjected to CFC and immediately received bilateral infusions of vehicle (0.9% NaCl) or ouabain (1 µM; 0.5 µL/side) into the BLA. Rats were euthanized 6 h later, and the hippocampus were dissected for western blot analysis of α1, α2, α3, and β1 isoforms. One outlier was identified and removed prior to statistical analysis. Data were expressed as mean ± SEM (*n* = 4–6/group). Unpaired *t*-test. *p* < 0.05 were considered statistically significant. Full-length blots including all samples and molecular weight markers are provided in the Supplementary Material

**Fig. 8:**
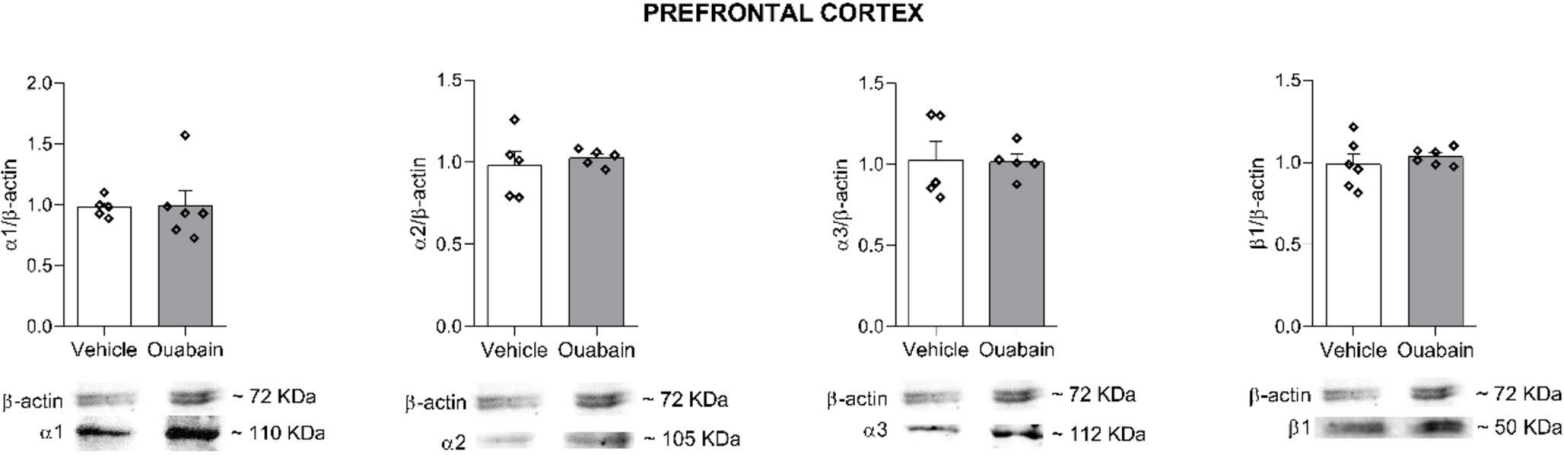
Ouabain infusion into the BLA does not alter Na^+^,K^+^-ATPase isoforms immunocontent in Prefrontal Cortex. Animals were subjected to CFC and immediately received bilateral infusions of vehicle (0.9% NaCl) or ouabain (1 µM; 0.5 µL/side) into the BLA. Rats were euthanized 6 h later, and the prefrontal cortex were dissected for western blot analysis of α1, α2, α3, and β1 isoforms. One outlier was identified and removed prior to statistical analysis. Data were expressed as mean ± SEM (*n* = 4–6/group). Unpaired *t*-test. *p* < 0.05 were considered statistically significant. Full-length blots including all samples and molecular weight markers are provided in the Supplementary Material

Western blot analysis was conducted on hippocampal and prefrontal cortex samples to assess protein levels of the relevant isoforms (Figs. 7 and 8, respectively). In both structures, no statistically significant differences were observed between groups for any isoform examined (*p* > 0.05).

### Oxidative Stress Analyses

**Fig. 9:**
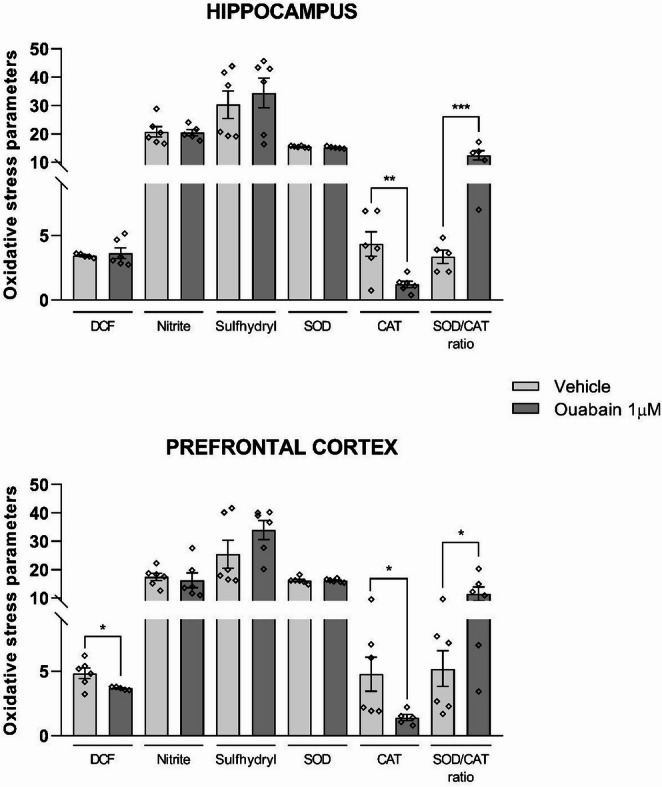
Ouabain infusion into the BLA altered oxidative stress parameters in the hippocampus and prefrontal cortex. Animals underwent CFC and immediately received bilateral infusions of vehicle (0.9% NaCl) or ouabain (1 µM; 0. 5 µL/side) into the BLA. Six hours later, the hippocampus and prefrontal cortex were collected for analysis of DCF levels, sulfhydryl content, nitrite levels, CAT activity, and SOD activity. Seven outliers were identified and removed prior to statistical analysis (4,9%). Data are expressed as mean ± SEM (*n* = 5–6/group). Unpaired *t*-test. *p* < 0.05 were considered statistically significant

Oxidative stress parameters were measured in the hippocampus and prefrontal cortex following ouabain infusion (Fig. 9). DCF fluorescence analysis indicated a reduction in reactive species levels in the prefrontal cortex (t = 2.495, df = 9, *p* = 0.0341), with no significant change observed in the hippocampus (*p* > 0.05). No significant differences in sulfhydryl content or nitrite levels were detected (*p* > 0.05). Analysis of antioxidant enzyme activities showed that SOD activity did not differ significantly in either brain region (*p* > 0.05). In contrast, CAT activity was lower in both the prefrontal cortex (t = 2.283, df = 9, *p* = 0.0483) and the hippocampus (t = 3.171, df = 10, *p* = 0.0100). This alteration resulted in an elevated SOD/CAT ratio in both regions (prefrontal cortex: t = 2.249, df = 10, *p* = 0.0483; hippocampus: t = 5.112, df = 8, *p* = 0.0009).

### Inflammatory Cytokines Expression

**Fig. 10:**
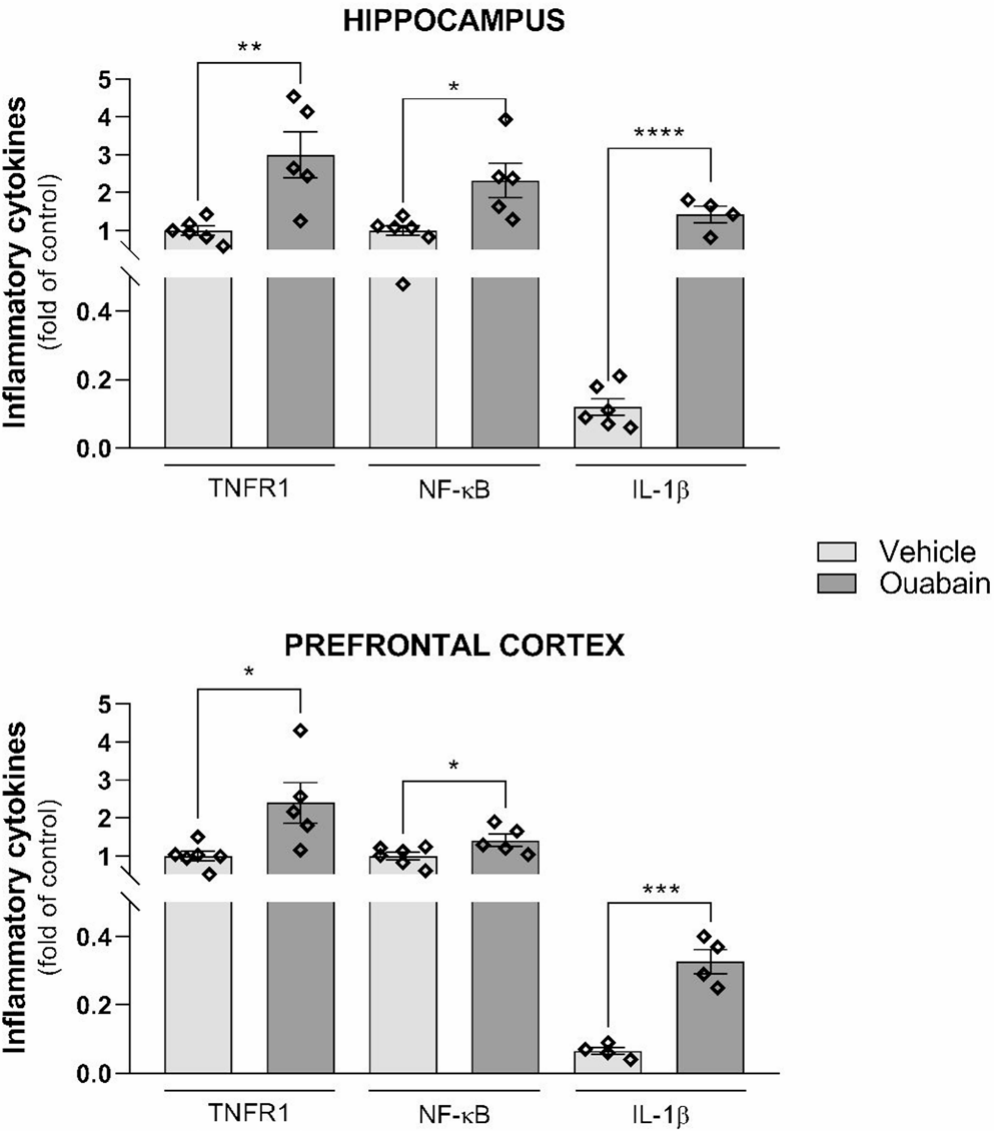
Ouabain infusion into the BLA increases the expression of pro-inflammatory cytokine genes in the hippocampus and prefrontal cortex. Animals underwent CFC and immediately received bilateral infusions of vehicle (0.9% NaCl) or ouabain (1 µM; 0.5 µL/side) into the BLA. Animals were euthanized after 6 h and the hippocampus and prefrontal cortex were dissected for RT-PCR analysis of TNFR1, NF-κB, and IL-1β mRNA levels. Ct value of control samples ranged from 31.62 to 35.89 for TNFR1, 28.99 to 33.99 for NF-κB, and 32.43 to 37.00 for IL-1β. Three amplifications were unsuccessful, and seven samples were identified as outliers and excluded from the statistical analysis (10%). Data are expressed as mean ± SEM (*n* = 4–6/group). Unpaired *t*-test. *p* < 0.05 were considered statistically significant

Given the observed alterations in redox parameters, pro-inflammatory cytokine expression was evaluated (Fig. 10). Six hours after infusion, TNFR1 mRNA levels were elevated in the ouabain-treated group in both the hippocampus (t = 3.609, df = 9, *p* = 0.0057) and the prefrontal cortex (t = 2.800, df = 9, *p* = 0.0207). Similarly, NF-κB mRNA was upregulated in the ouabain-treated group in the hippocampus (t = 3.069, df = 9, *p* = 0.0134) and the prefrontal cortex (t = 2.280, df = 9, *p* = 0.0486). IL-1β mRNA expression was also increased in the ouabain-treated group in the hippocampus (t = 7.484, df = 8, *p* < 0.0001) and the prefrontal cortex (t = 7.240, df = 6, *p* = 0.0004).

## Discussion

The present study investigated the interplay between Na^+^,K^+^-ATPase function, neuroinflammation, oxidative imbalance, and the consolidation of fear memory following ouabain infusion into the BLA. We demonstrate that pharmacological inhibition of Na^+^,K^+^-ATPase in the BLA produces a robust impairment in contextual fear memory consolidation and triggers a cascade of biochemical alterations in interconnected brain structures. These findings underscore the functional coupling between amygdalar Na^+^,K^+^-ATPase activity and downstream molecular events in the hippocampus and prefrontal cortex, two brain regions essential for the formation and stabilization of aversive memories.

Behaviorally, ouabain infusion into the BLA significantly reduced freezing behavior during the memory test, consistent with a deficit in consolidation rather than acquisition or retrieval. This aligns with extensive evidence indicating that the BLA orchestrates emotional memory consolidation by modulating several interconnected structures, including the hippocampus and medial PFC (Maren et al. 2013; Phelps and LeDoux 2005). Notably, although ouabain was infused locally into the BLA, changes in Na^+^,K^+^-ATPase activity were detected only in the hippocampus (6 h) and PFC (24 h), but not in the amygdala itself. This reinforces the concept that BLA perturbations can propagate through long-range circuits, altering metabolic and transcriptional states in distant, memory-related regions.

To understand this circuit-level propagation, it is critical to consider the established role of the BLA in integrating stress and memory signals. Previous evidence demonstrates that effective consolidation of aversive memories depends on intact bidirectional communication between the BLA and the medial prefrontal cortex, critically engaging ERK/MAPK signaling cascades during the post-training period (Roozendaal et al. 2009). The BLA integrates peripheral stress-related signals, including glucocorticoids and catecholamines, and influences memory-related structures through noradrenergic projections to the hippocampus and neocortex, thereby modulating memory consolidation and expression (McGaugh and Roozendaal 2009). Hippocampal CA1 and PrL regions are known to contribute preferentially to contextual representation, discrimination, and the regulation or expression of fear responses at later stages, rather than serving as primary loci for the initial consolidation process (Rudy 2009; Sotres-Bayon and Quirk 2010). Thus, the absence of an effect on memory consolidation when ouabain is infused into CA1 and PrL immediately after training may reflect a temporally restricted role of these regions in this specific CFC paradigm, rather than a lack of involvement per se. In this framework, our findings support the notion that the BLA operates as a critical gatekeeper during early consolidation (Nathan et al. 2004).

In parallel, it is well established that behavioral paradigms involving aversive learning, such as contextual fear conditioning, robustly activate the hypothalamic–pituitary–adrenal (HPA) axis. In this context, studies using chronic stress paradigms have shown that functional inactivation of the BLA attenuates stress-induced cognitive and behavioral impairments and normalizes HPA-axis–related outcomes, highlighting the role of the BLA as a key interface between stress physiology and higher-order cognitive circuits (Tripathi et al. 2017, 2019a, b). Notably, these manipulations were typically performed prior to learning and focused on recognition or spatial memory paradigms. In contrast, our findings demonstrate that transient disruption of BLA function specifically during the post-training consolidation window is sufficient to alter downstream molecular responses in interconnected regions and impair fear memory consolidation, underscoring a time- and circuit-dependent role of BLA signaling in coordinating stress-related and mnemonic processes.

The memory impairment induced by ouabain, which is not rescued by D-serine co-administration, can be understood in light of the direct biophysical consequences of Na⁺,K⁺-ATPase inhibition on synaptic function. This finding suggests that enhancing NMDA receptor co-agonist availability may not be an effective mechanism to overcome the ionic disturbances resulting from pump inhibition. A primary mechanism likely involves ouabain-induced collapse of Na⁺ and K⁺ gradients, leading to sustained membrane depolarization and reversal of the Na⁺/Ca²⁺ exchanger, thereby promoting pathological Ca²⁺ influx (Rose and Ransom 1997; Yu 2003; Kinoshita et al. 2022). Within this altered synaptic environment, NMDA receptors operate under conditions of elevated basal Ca²⁺ levels and disrupted electrochemical driving forces. As a result, receptor activation may shift from generating a spatially and temporally restricted Ca²⁺ signal that supports plasticity to producing a sustained and poorly compartmentalized Ca²⁺ load (Xiong et al. 1999; Sibarov et al. 2012). In this context, the failure of D-serine to restore consolidation becomes mechanistically coherent. Although D-serine facilitates NMDA receptor gating via high-affinity binding to the GluN1 co-agonist site, such facilitation under conditions of ionic dysregulation would be unlikely to re-establish plasticity and may instead exacerbate maladaptive Ca²⁺-dependent signaling (Coyle et al. 2020; Lisman 2001). Thus, the D-serine-insensitive impairment observed is more consistent with a fundamental disruption of the ionic and signaling environment required for NMDAR-dependent plasticity than with insufficient receptor co-agonism per se.

This dysfunction, however, extends beyond a purely cellular disturbance, engaging specific signaling pathways that are critically implicated in synaptic plasticity. The Na⁺,K⁺-ATPase-dependent signaling contributes to the regulation of synaptic plasticity through modulation of MAP kinase pathways. At 1 µM, ouabain induces transient ERK1/2 activation together with sustained p38 phosphorylation without affecting neuronal viability, a signaling profile consistent with adaptive plasticity-related processes (Lopachev et al. 2016). Although cell culture studies suggest that this concentration preferentially targets the ouabain-sensitive α3 isoform and modulates NMDA receptor function (Akkuratov et al. 2015), our findings indicate that ouabain at the same dose is associated with changes in α1 and β1 isoforms expression. These observations likely reflect the greater cellular complexity and network integration inherent to *in vivo* conditions, in which systemic and circuit-level mechanisms may shape Na⁺,K⁺-ATPase regulation beyond isoform-specific effects, supporting the use of 1 µM ouabain as a physiologically relevant dose to investigate plasticity-related signaling.

These observed changes in subunit expression are consistent with the dynamic, region-specific alterations in enzymatic activity we measured following ouabain infusion. Biochemically, Na^+^,K^+^-ATPase activity displayed a temporally and regionally specific pattern following ouabain administration. The early reduction in hippocampal activity (6 h) coincided with unchanged gene expression and protein levels of catalytic and regulatory subunits, suggesting direct pharmacological inhibition or acute ionic/metabolic effects. By contrast, the later increase in PFC enzymatic activity (24 h) was accompanied by elevated expression of α1 and β1 isoforms. Similar compensatory upregulation of Na^+^,K^+^-ATPase subunits following pump inhibition has been reported in astrocytes (Hosoi et al. 2002) and may reflect a homeostatic attempt to restore ionic balance. The β1 isoform, known to modulate ion affinity and turnover rate (Xu 2011), may contribute to the increased Na^+^,K^+^-ATPase activity observed at the later time point.

Regarding oxidative stress, ouabain induced a paradoxical reduction in DCF fluorescence in the PFC despite decreasing CAT activity in both brain structures. This pattern likely reflects methodological limitations of DCF for detecting compartmentalized ROS rather than a true reduction in oxidative burden (Kalyanaraman et al. 2012). Decreased CAT activity implies a reduced capacity to detoxify hydrogen peroxide, consistent with enhanced susceptibility to oxidative damage and initiation of pro-inflammatory signal cascades. The increased SOD/CAT ratio further suggests an imbalance favoring H_2_O_2_ accumulation, which can activate NF-κB and TNFR1 pathways (Sies 2017). Such redox imbalance may have contributed to the observed impairment in memory consolidation, as oxidative stress interferes with the phosphorylation of key long-term potentiation (LTP)-related proteins, including CaMKII and CREB (Picón-Pagès et al. 2022).

We observed marked upregulation of TNFR1, NF-κB and IL-1β in the hippocampus and PFC. These cytokines are key mediators of neuroinflammation and are well known to compromise synaptic plasticity by reducing LTP and favoring long-term depression (LTD), thereby exerting a negative impact on mnemonic and executive functions mediated by the hippocampus and prefrontal cortex (McCoy and Tansey 2008; Yirmiya and Goshen 2011; Di Filippo et al. 2008). The regional specificity of this response aligns with recent evidence that BLA stimulation can induce neuroinflammatory responses in downstream structures (Adkins et al. 2023). Given that all animals were subjected to contextual fear conditioning, the amplified cytokine expression observed here likely reflects ouabain-induced modulation rather than behavioral stress alone.

Taken together, these findings indicate that Na^+^,K^+^-ATPase inhibition in the BLA initiates a cascade of ionic, oxidative, and inflammatory alterations in circuit-related brain regions crucial for fear memory consolidation. By disrupting ionic homeostasis, dampening antioxidant defenses, and enhancing pro-inflammatory transcriptional programs, ouabain undermines molecular processes required for stable memory formation.

## Conclusions and Study Limitations

**Fig. 11:**
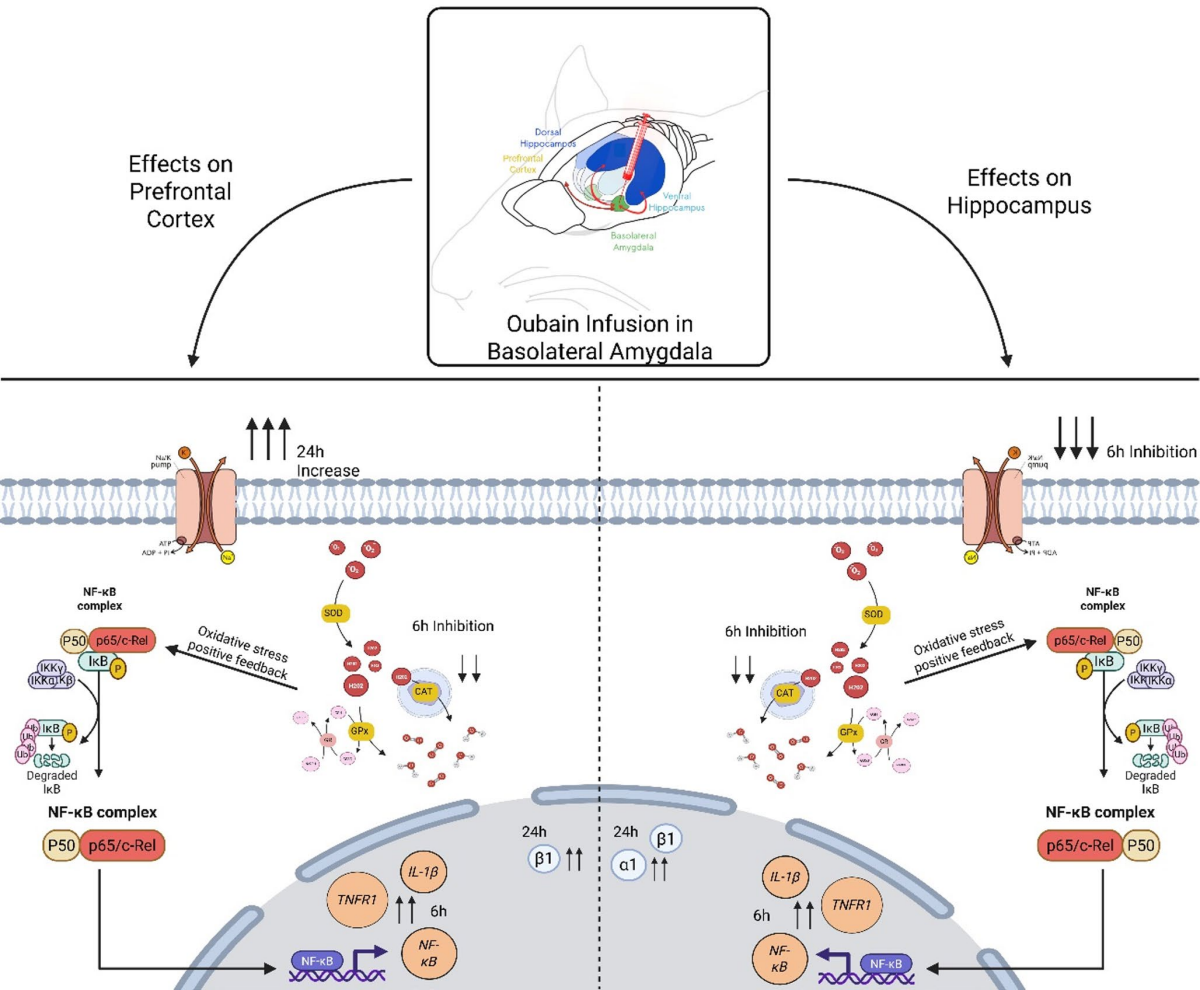
Schematic overview of the time- and region-dependent effects of ouabain infusion into the BLA. Local ouabain administration differentially modulates prefrontal cortex and hippocampal signaling, inducing early (6 h) reduction in Na⁺,K⁺-ATPase activity, oxidative stress and activation of the NF-κB pathway with increased pro-inflammatory markers (IL-1β, TNFR1). At later time point (24 h), a compensatory upregulation of Na⁺,K⁺-ATPase α1 and β1 isoforms and increased activity are observed, suggesting adaptive responses to the initial ionic and redox imbalance

Our findings demonstrate that Na^+^,K^+^-ATPase plays a critical role in the consolidation of fear memory and that its inhibition in the BLA disrupts not only local functions but also induces region-specific alterations in the hippocampus and prefrontal cortex. Ouabain infusion produced time-dependent changes in Na^+^,K^+^-ATPase activity, increased expression of α1 and β1 isoforms, altered redox homeostasis, and promoted pro-inflammatory gene expression (Fig. 11). These data support the notion that Na^+^,K^+^-ATPase dysfunction triggers neurotoxic processes involving oxidative stress and inflammation that may compromise neural plasticity. Understanding how Na^+^,K^+^-ATPase regulates these pathways may provide mechanistic insights relevant to neuropsychiatric and neurodegenerative disorders.

This study has limitations. Although robust alterations in inflammatory and oxidative stress parameters were observed, these changes cannot be attributed as causal factors underlying the behavioral impairments, but rather should be interpreted as being associated with the observed outcomes. Future studies employing pharmacological or genetic modulation of redox and inflammatory pathways will be necessary to directly test causality. Moreover, protein expression levels and post-translational modifications, particularly those regulating redox-sensitive signaling pathways and inflammatory cascades, were not assessed. Additionally, only male rats were evaluated, and sex differences in neuroinflammation and Na^+^,K^+^-ATPase regulation warrant future investigation.

## Supplementary information

Below is the link to the electronic supplementary material.

Supplementary File 1 (DOCX 2.36 MB)

## Data Availability

The dataset generated and analyzed during the current study are available from the corresponding author upon reasonable request.
