# Supplementary material for: Regional Crosstalk Between the Amygdala, Hippocampus, and Prefrontal Cortex Following Na+,K+-ATPase Inhibition by Ouabain
Source: Neurotox Res. 2026 May 7;44(3):25. doi: 10.1007/s12640-026-00800-7 (PMC13152888; doi:10.1007/s12640-026-00800-7)
Supplement: Supplementary file 1 — (DOCX 2.36 MB) [file 12640_2026_800_MOESM1_ESM.docx]

**Supplementary data**


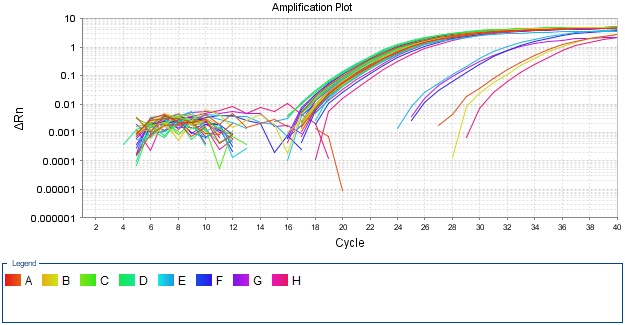


**Fig S1**. Amplification of the gene encoding α1


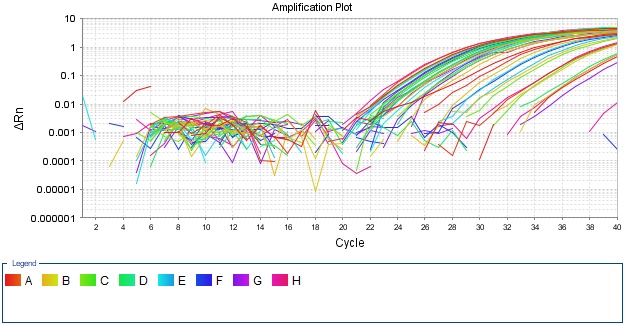


**Fig S2**. Amplification of the gene encoding α2


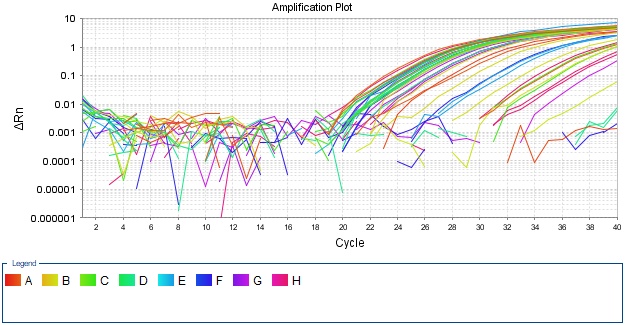


**Fig S3**. Amplification of the gene encoding α3


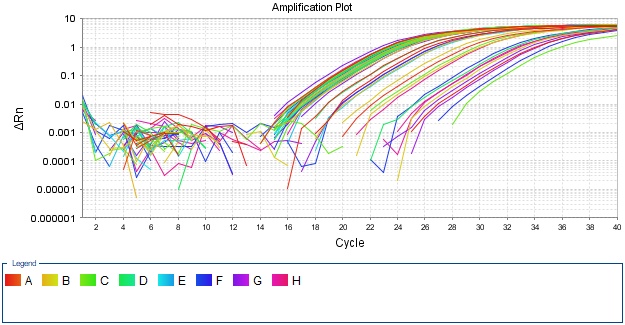


**Fig S4**. Amplification of the gene encoding β1


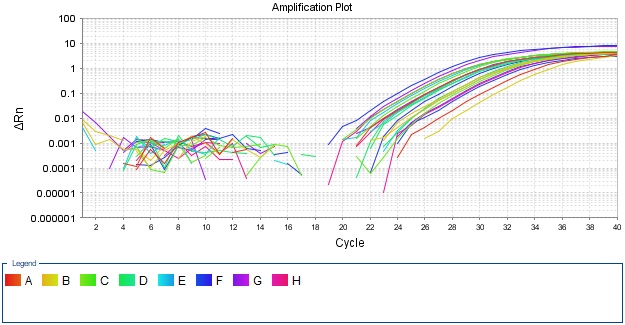


**Fig S5**. Amplification of the gene encoding NF-κB


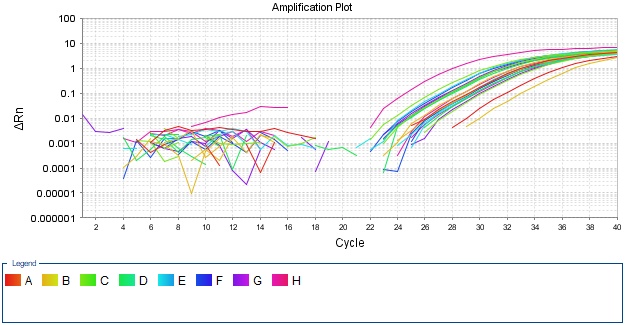


**Fig S6**. Amplification of the gene encoding TNFR1


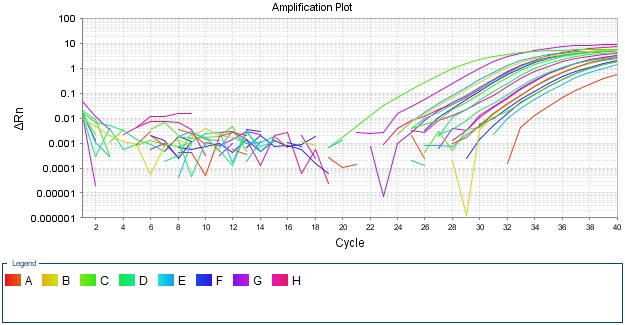


**Fig S7**. Amplification of the gene encoding IL-1β


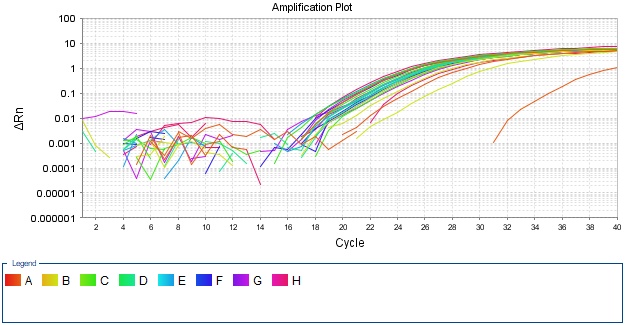


**Fig S8**. Amplification of the gene encoding β-actin


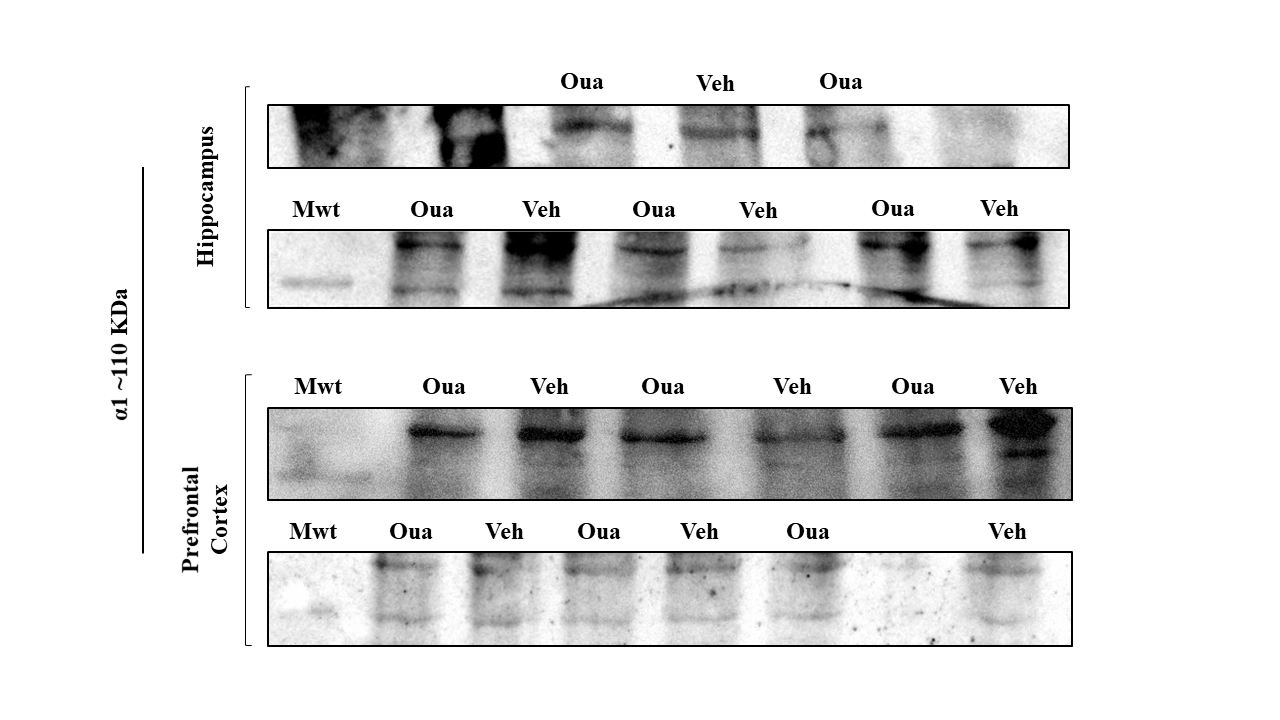
**
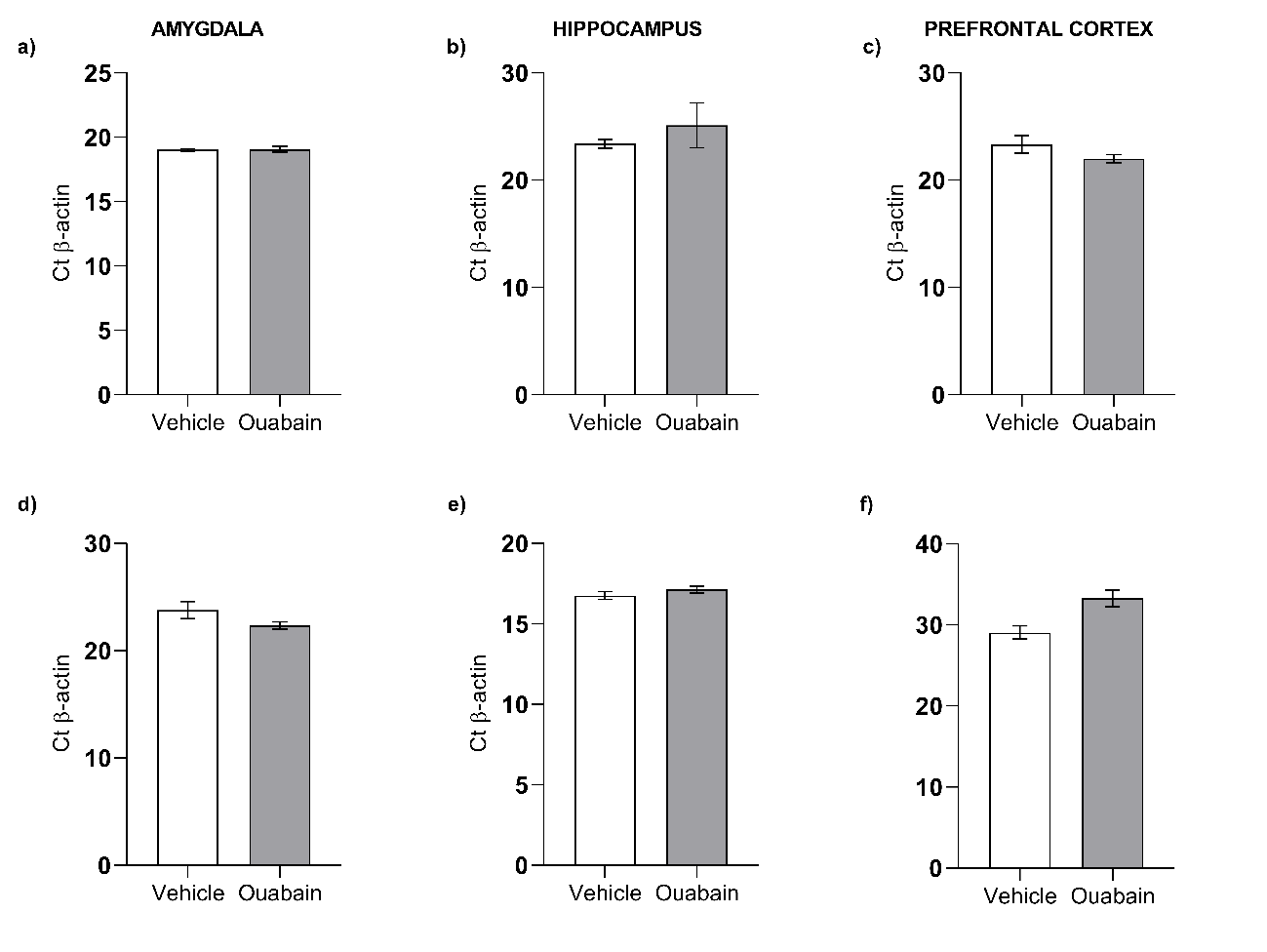
Fig S9. Ct values of β-actin for the 6 h and 24 h cohorts.** Graphs a - c show Ct values from samples collected 6 hours after infusion, and graphs d - f show Ct values from the 24-hour cohort. No differences were observed between vehicle and ouabain-treated groups. Unpaired *t*-test.

**Fig S10. Representative full-length western blot images of Na⁺,K⁺-ATPase α1 in hippocampus and prefrontal cortex.** Full, uncropped western blot membranes are shown for hippocampus (upper panels) and prefrontal cortex (lower panels), including all experimental samples from vehicle (Veh) and ouabain-treated (Oua) groups. Molecular weight markers (Mwt) are indicated on the left.


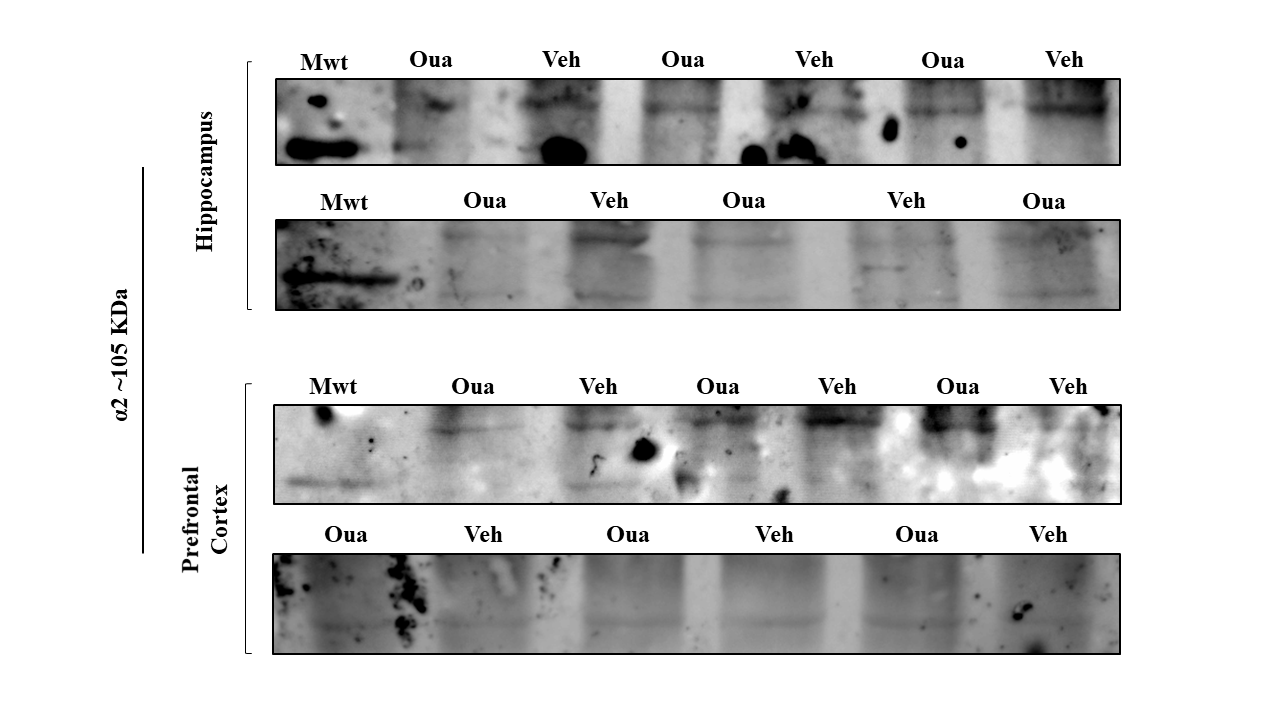


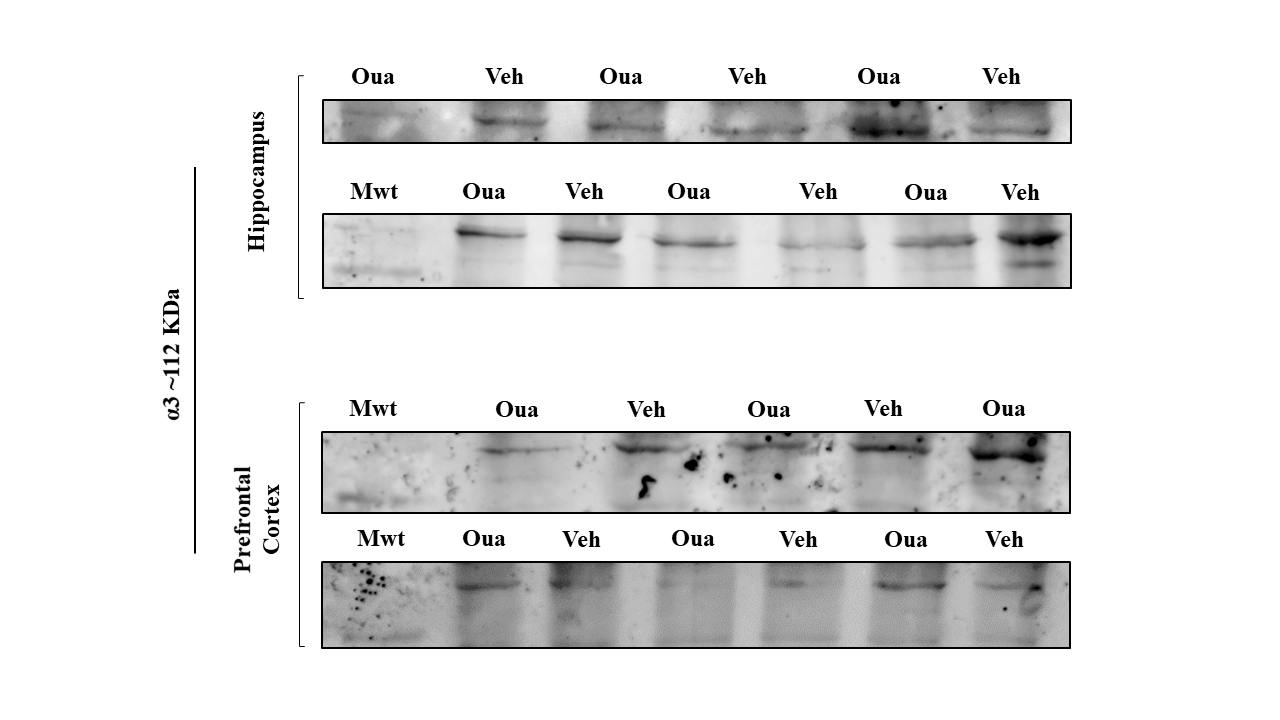
**Fig S11. Representative full-length western blot images of Na⁺,K⁺-ATPase α2 in hippocampus and prefrontal cortex.** Full, uncropped western blot membranes are shown for hippocampus (upper panels) and prefrontal cortex (lower panels), including all experimental samples from vehicle (Veh) and ouabain-treated (Oua) groups. Molecular weight markers (Mwt) are indicated on the left.

**Fig S12. Representative full-length western blot images of Na⁺,K⁺-ATPase α3 in hippocampus and prefrontal cortex.** Full, uncropped western blot membranes are shown for hippocampus (upper panels) and prefrontal cortex (lower panels), including all experimental samples from vehicle (Veh) and ouabain-treated (Oua) groups. Molecular weight markers (Mwt) are indicated on the left.

**Fig S13**. **Representative full-length western blot images of Na⁺,K⁺-ATPase β1 in hippocampus and prefrontal cortex.** Full, uncropped western blot membranes are shown for hippocampus (upper panels) and prefrontal cortex (lower panels), including all experimental samples from vehicle (Veh) and ouabain-treated (Oua) groups.
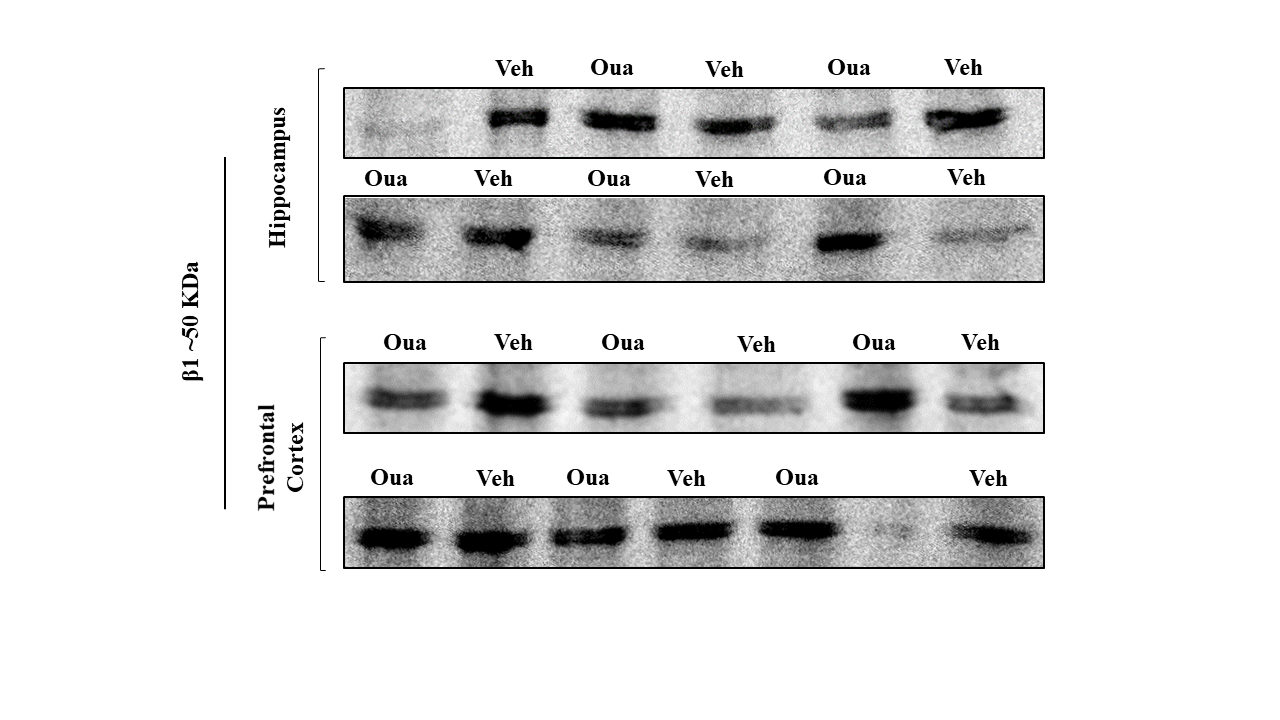


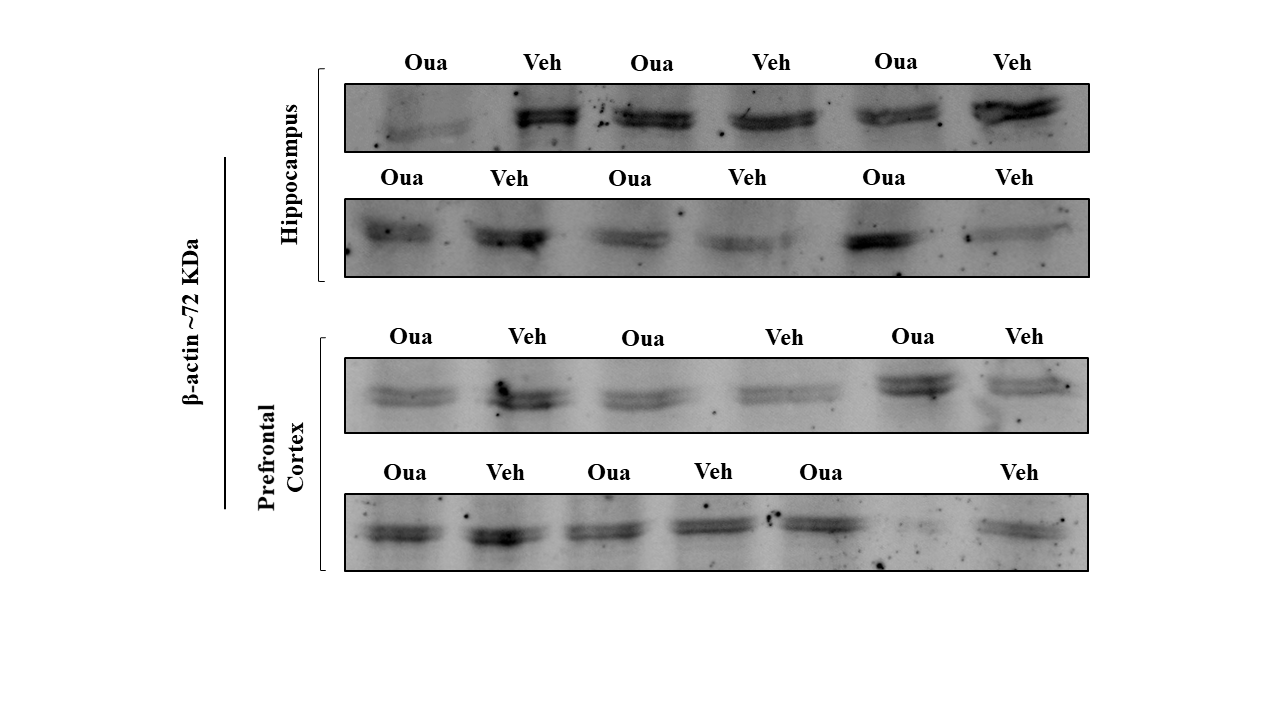


**Fig S13 Representative full-length western blot images of β-actin (42 kDa) in hippocampus and prefrontal cortex.** Full, uncropped western blot membranes are shown for hippocampus (upper panels) and prefrontal cortex (lower panels), including all experimental samples from vehicle (Veh) and ouabain-treated (Oua) groups. β-actin was used as a loading control and was detected on the same membranes after stripping and reprobing. All samples were processed under identical experimental conditions.
